# Alexandra General Hospital in Athens: Historical Connections to the Greek Royal Family

**DOI:** 10.7759/cureus.32985

**Published:** 2022-12-27

**Authors:** Spyros N Michaleas, Konstantinos Laios, Ioannis Nikolakakis, Ioannis Dimitriadis, Marianna Karamanou, Effie Poulakou-Rebelakou

**Affiliations:** 1 Department of History of Medicine and Medical Ethics, National and Kapodistrian University of Athens School of Medicine, Athens, GRC

**Keywords:** princess alexandra of greece, obstetrics-gynaecological hospital, nikolaos louros, russia, maternity hospital

## Abstract

Princess Alexandra of Greece (1870-1891), the eldest daughter of King George I of Greece (1845-1913), was known as the “beloved daughter of the Athenians”. Her death at the age of 21 in 1891 due to a pregnancy complication caused nationwide grief. To honour her, the Alexandra Maternity Hospital in Athens was named in her memory. Affiliated with the University of Athens, Alexandra Maternity Hospital researches pregnancy and newborn care, including complications and maternal mortality. Today, the hospital contains various clinical and laboratory departments providing patients with exceptional health care.

## Introduction and background

In 1834, Greece issued a royal decree founding the Health Council (*Iatrosynedrio*), an institution responsible for public health management [1]. Iatrosynedrio operated in Greece until the mid-1960s, leading a wave of major health reforms and facility construction. These facilities, funded mainly by donations, included the Alexandra Public Maternity Hospital, an exemplary hospital for mother and newborn care, as well as a hub of scientific research in obstetrics and gynaecology connected to the Medical School of the University of Athens and led by Nikolaos Louros (1898-1986), its founding director and professor [2,3]. Alexandra Public Maternity Hospital was named after beloved Princess Alexandra, the eldest daughter of King George I of Greece, who died during her second pregnancy in 1891. Today, the hospital is known as the General Hospital of Athens Alexandra, with services specializing in gynaecology and cardiology [4].

## Review

Origins of Alexandra General Hospital

After the Greek War of Independence (1821-1832) against the Ottomans, the newly formed Greek State faced a variety of issues, many of them related to public health services. The Greek Health Council Iatrosynedrio lobbied for an obstetrics and gynaecology clinic to protect mothers and infants. Initial attempts to establish and operate a privately funded maternity hospital failed [2]. Finally, around 1835, the Greek Government under King Otto established the first maternity hospital in Athens [2]. Then, in 1836, a government-funded Public Maternity Hospital was established at the University of Athens. Its main purpose was to educate midwives, train medical students, and provide free care to new mothers and their infants.

In 1892, the rector of the University of Athens, Pavlos Ioannou (1824-1897), suggested naming the hospital Alexandra Maternity Hospital in memory of Princess Alexandra of Greece and Denmark (1870-1891). According to the articles of association, its purpose would be to care for needy patients and provide scientific assistance to the University of Athens Medical School. By 1899, the university has raised enough funds to purchase 10,000 square metres of land next to Aretaieio Hospital, owned by the Monastery of Asomaton [3]. That same year, the Monastery granted an additional 530 square metres, but construction of the new hospital was stalled due to financing issues, so the staff continued to work in rented buildings [4].

In 1908, the Minister of Interior, in a letter to Konstantinos Louros (1864-1957), director of the Alexandra Maternity Hospital, described the terrible conditions at the Public Maternity Hospital. The letter appeared to have worked because soon after in 1909, upgrading and renovation work resumed. The new ward had 50 beds and rooms for delivery, operating, newborn care, and staff, as well as baths, toilets, and an isolation ward with 10 beds.

Then, in 1915, the institution moved to a new location on Akadimias Street that belonged to Ioannis Eftaxias (1848-1927), rector of the University of Athens [4]. This two-storey building had a large garden but significant deficiencies. In 1917, a competition was held to find a new home for the hospital, preferably in a modern building with a classroom for students and midwives. However, Ioannis Eftaxias won the competition, though he promised to build a classroom and make the necessary repairs [4]. In 1934, another attempt was made to construct a new building next to Aretaieio Hospital in Athens. Foundation work started immediately but ceased in 1936 when the University of Athens ran out of funding. By 1938, 70-bed Alexandra Public Maternity Hospital was still housed in the building belonging to Ioannis Eftaxias.

**Figure 1:**
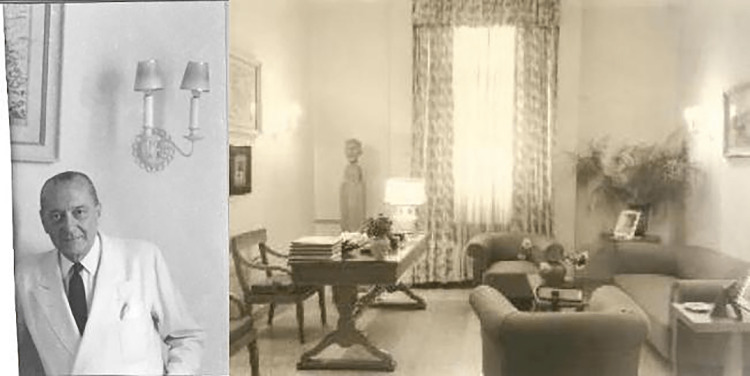
Professor Nikolaos Louros and his office Free open access figure. No attribution is required. Credit: http://hosp-alexandra.gr

Finally, in 1941, the facility was relocated to a building on Zalokosta Street in Athens. Minister of Health Alexandros Koryzis (1885-1941) had appointed Nikolaos Louros, professor and director of the hospital, to draft a new operational program (Figure 1) [5].

**Figure 2:**
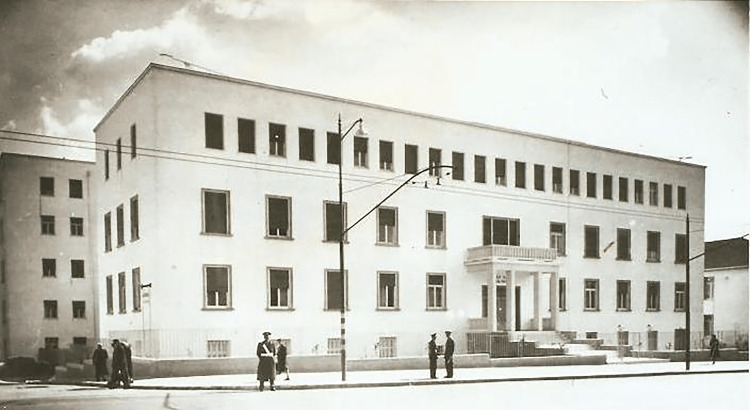
Alexandra Maternity Hospital in 1954 Free open access figure. No attribution is required. Credit: http://hosp-alexandra.gr

In 1944, the University of Athens and the Public Maternity Hospital ratified a contract granting the State the right to build a suitable building for the installation and operation of the Public Maternity Hospital [4]. With financial support from the US-funded European Recovery Program (Marshall Plan), Professor Nikolaos Louros and Queen Frederica of Greece (1917-1981) worked to complete construction (Figure 2).

Thus, more than 60 years after its initial founding and interrupted by two World Wars and severe financial instability in Greece, Alexandra Public Maternity Hospital at the University of Athens became a reality [2].

**Figure 3:**
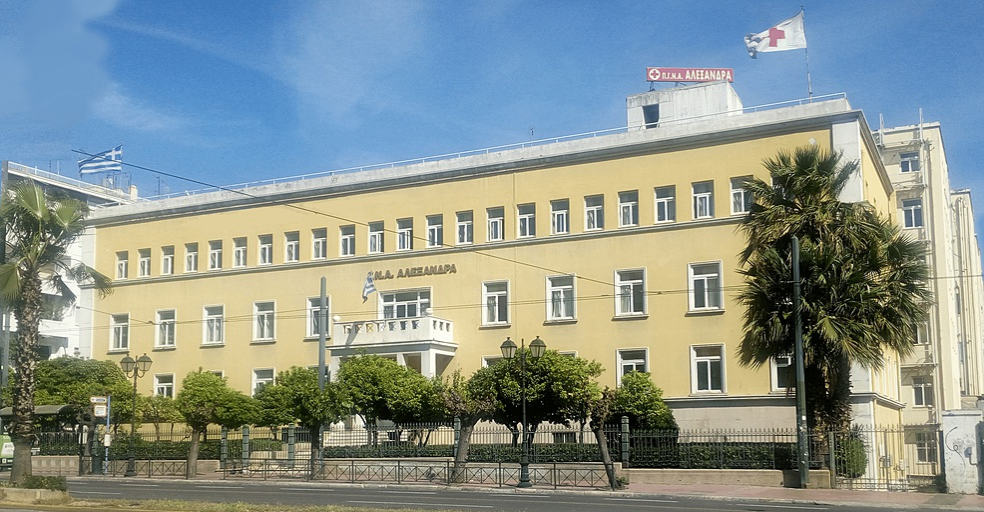
General Hospital of Athens Alexandra, on Vasilissis Sofias Avenue, in 2022 Free open access figure. No attribution is required. Credit: http://hosp-alexandra.gr

The inauguration took place on December 10, 1954, and was attended by representatives of the Greek State, the University of Athens, and several other officials. Director Louros described it as an exemplar of obstetrics and gynaecology care, emphasizing its goal as a hospital centre for the protection of mother and child and as a centre of scientific research and education. Moreover, the hospital used state-of-the-art equipment such as roentgen and isotopes in the Radiology Department [5]. From 1954 to 1968, Louros assumed the direction of the Maternity Hospital and the First Obstetrics and Gynaecology Clinic of the University of Athens, which was housed inside the Alexandra building [5]. In 1955, the institution became a legal entity under public law. Now known as the General Hospital of Athens Alexandra, it is located on Vasilissis Sofias Avenue (Figure 3). It has a capacity of more than 350 beds, with over 1,000 staff members and several clinical and laboratory departments, including obstetrics and gynaecology, medical therapeutics, and oncology [2-4].

Princess Alexandra of Greece and Denmark (1870-1891)

Princess Alexandra was born on August 30, 1870, in the royal mansion Mon Repos on the island of Corfu. She was the third of eight children of King George I of Greece (1845-1913) and his wife, Russian-born Grand Duchess Olga Constantinovna (1851-1926). As the eldest daughter of the royal family, Alexandra was given the title of Princess of Greece [6]. Her beauty, kind personality, and charming manners steeped in the traditions of Greek culture made her popular among both family and visitors. Known as Alex by those close to her, the princess had friends inside and outside the royal courtyard. Thus, it was not long before she was given the title of “the beloved daughter of the Athenians” [6,7].

**Figure 4:**
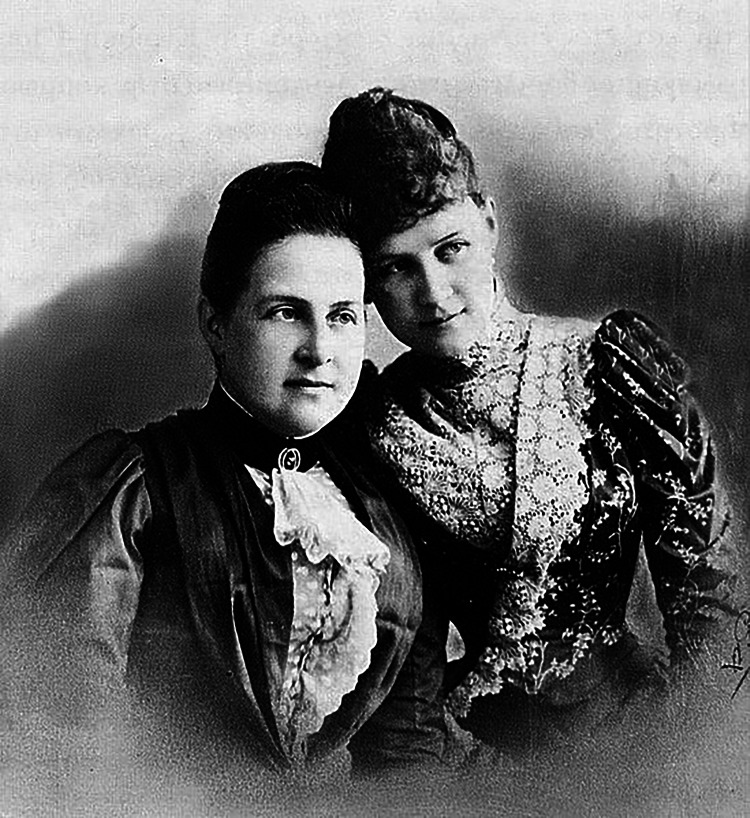
Princess Alexandra with her mother, Queen Olga, c1888 Free open access figure. No attribution is required. Credit: http://royal-splendor.blogspot.com/2021/08/alexandra-of-greece-grand-duchess-of-russia.html

As the eldest daughter of an elite family, Princess Alexandra received a proper education, studying Greek, English, French, and German, as well as arithmetic, music, painting, and horseback riding. Her mother, Queen Olga, was deeply religious and instilled a religious upbringing in all of her children (Figure 4). Alexandra also often accompanied her father, King George I of Greece, on his sea voyages on the royal ship Amphitrite [6,7].

Queen Olga remained attached to her Russian homeland and dreamed of marrying her daughters to Russian noblemen. For this reason, she chose her first cousin and son of Tsar Alexander II Romanov (1860-1919), Grand Duke Paul Alexandrovich (1860-1919), to wed her daughter Alexandra [6,7].

On May 20, 1889, at age 19, the beloved princess left Greece for Russia, where she married Grand Duke Paul on June 17, 1889, in the royal winter palace in Saint Petersburg. The ceremony was celebrated with great formality and luxury [7]. However, Alexandra eschewed the traditional wedding tiara and opted instead to wear a wreath made of myrtle from Tatoi Palace, the Greek royal estate. Upon her marriage to the Grand Duke, she was called Alexandra Georgievna and took the title of Grand Duchess of Russia. Though popular among the Russian Imperial Court for her wit and intellect, Alexandra did not like living there and she missed her homeland [6,7].

On April 18, 1890, Alexandra gave birth to their first child, Maria. Grand Duke Paul was serving in the constitution, so Alexandra was often left alone at the palace and fell into melancholy. When she became pregnant again the next year, everyone awaited the crown prince. Alexandra’s health had weakened since her previous pregnancy, however. In her seventh month of pregnancy, she visited the estate of Paul’s brother, Grand Duke Sergei (1857-1905) and his wife, Elisabeth of Hesse (1864-1918), in Ilinskoye, a village near Moscow. While stepping onto a boat on the Moskva River, Alexandra fell. The next day, she started to labour prematurely and gave birth to Grand Duke Dmitri Pavlovich (1891-1942) [6]. Unfortunately, Alexandra lapsed into a coma immediately after birth due to eclampsia and postpartum fever. She died six days later on September 24, 1891, at the age of 21. The tragic news spread in a wave of sadness across Russia and Greece. Boulevards and squares were named in her honour, as well as the Alexandra Public Maternity Hospital [6]. Alexandra was buried at the Cathedral of Saints Peter and Paul in Saint Petersburg until around 1940, when the King of Greece George II (1890-1947), Alexandra’s nephew, mediated for the return of her remains to Greece to be interred at the Royal Cemetery at Tatoi Palace [6].

About a decade after Alexandra’s death in 1902, her widow, Grand Duke Paul, was banished when he married morganatically the commoner Olga Valeriánovna Karnóvich, with whom he had three more children. Grand Duke Dmitri and Grand Duchess Maria were adopted by their paternal uncle, Grand Duke Sergei, and his wife, Elisabeth [6]. Dmitri later became famous for his involvement in the murder of Grigori Yefimovich Rasputin (1869-1916), the faith healer and spiritual advisor of the Russian imperial family. He and Prince Felix Felixovich Yusupov (1887-1967) were suspicious of Rasputin’s maleficent influence on the imperial couple and believed his death would solve all the difficulties of the royal family and the country. Dmitri had been considered a potential husband for Grand Duchess Olga Nikolaevna of Russia (1895-1918), daughter of Tsar Nicholas II of Russia (1894-1917) and Tsaritsa Alexandra Feodorovna (1872-1918). When the Tsar found out that a member of the royal family had been involved in Rasputin’s grisly murder (which involved poisoning, gunshot, and ultimately drowning), he sent Dmitri into exile, an action that saved his life [8,9].

Dmitri’s sister, Maria Pavlovna, travelled to Paris, where she worked in the fashion industry. She founded a business called Kitmir, which specialized in bead and sequin embroidery. Maria worked for Gabrielle Bonheur Coco Chanel (1883-1971), with whom her brother Dmitri was romantically involved for a short time. Dmitri introduced her to Ernest Beaux, the Tsars’ former perfumer, who created Chanel No. 5, the brand’s iconic perfume [10,11].

## Conclusions

In 1830, the Greek State recognized a dire need for organized public health services. One of its priorities was the establishment of a university-based public maternity hospital to protect mothers and their infants. After several attempts, the Alexandra Maternity Hospital, today known as the General Hospital of Athens Alexandra, broke ground in 1954. The tragic destiny of Princess Alexandra of Greece and Denmark, who died due to an obstetrical complication, led to the creation of the hospital, which remains an example of research excellence and high-quality healthcare services in modern Greece.
